# Distinct Advantages of Circumferential Notch Tensile (CNT) Testing in the Determination of a Threshold for Stress Corrosion Cracking (K_ISCC_)

**DOI:** 10.3390/ma14195620

**Published:** 2021-09-27

**Authors:** R. K. Singh Raman, Rhys Jones

**Affiliations:** 1 Department of Mechanical & Aerospace Engineering, Monash University, Melbourne, VIC 3800, Australia; rhys.jones@monash.edu; 2 Department of Chemical Engineering, Monash University, Melbourne, VIC 3800, Australia

**Keywords:** stress corrosion cracking (SCC), threshold stress intensity for SCC (K_ISCC_), circumferential notch tensile (CNT) testing, steel, magnesium

## Abstract

Stress corrosion cracking (SCC) is a vexing problem for load-bearing equipment operating in a corrosive environment in various industries, such as aerospace, chemical and mineral processing, civil structures, bioimplants, energy generation etc. For safe operation, effective maintenance and life prediction of such equipment, reliable design data on SCC (such as threshold stress intensity for SCC, i.e., K_ISCC_) are invaluable. Generating reliable K_ISCC_ data invariably requires a large number of tests. Traditional techniques can be prohibitively expensive. This article reviews the determination of K_ISCC_ using the circumferential notch tensile (CNT) technique, the validation of the technique and its application to a few industrially relevant scenarios. The CNT technique is a relatively recent and considerably inexpensive approach for the determination of K_ISCC_ when compared to traditional techniques, viz., double-cantilever beam (DCB) and compact tension (CT) that may be fraught with prohibitive complexities. As established through this article, the CNT technique circumvents some critical limitations of the traditional techniques.

## 1. Introduction

Stress corrosion cracking (SCC), i.e., a premature, sudden and sometimes catastrophic fracture, results from the synergy of corrosive medium and tensile stress. Because SCC is among the most catastrophic and dangerous types of degradation of metals/alloys caused by corrosion, it is a critical material selection criterion for load-bearing equipment designed for corrosive conditions. SCC is a critical concern because [1,2,3]:

(a) It can occur at stresses even below the yield strength of the material;
(b) Cracks can grow undetected into leaks or, sometimes, sudden and catastrophic failures when the required synergy of stress and environment is present;
(c) A few localized and fine cracks may grow undetected to fracture, while the alloy surface may virtually appear free from corrosion;
(d) It is intriguing that sometimes a relatively less corrosive environment may be more deleterious for SCC.

Mechanism, monitoring and mitigation of SCC are of great research and commercial interests, since SCC is a critical concern for a vast number of industrial equipment operating in corrosive environment, and its timely circumvention is critical for safe plant operation and maintenance [3]. Sometimes, in-service monitoring of the susceptibility to SCC may be required for the remaining life assessment and safe operation, whereas there may be occasions when the characterisation of SCC of a failed equipment may assist in the mitigation of likely SCC of other operating members. Given the stochastic nature of SCC, a confirmation of the phenomenon and reliable implementation of mitigation measures may require a vast number of tests. Therefore, it is very attractive to have an accurate, inexpensive and rapid technique for the characterization of susceptibility to and the generation of design data for SCC.

U-bend and C-ring testing are used for in-service monitoring of SCC [2,3] but they may represent excessively high loadings. On the other hand, a commonly employed laboratory technique, slow strain rate testing (SSRT), only provides a ‘go, no-go’ type of assessment [2,3]. Designing equipment that handle corrosive conditions in service or life extension/prediction of such equipment requires data on the likelihood of existing surface defects/cracks to undergo corrosion-assisted growth. This requires the determination of the threshold of the stress intensity factor (K_I_) for growth of a pre-existing surface defect/crack [1]. However, in a corrosive environment, K_I_ can be influenced by the corrosion at the tip of a crack. Therefore, the threshold of the stress intensity factor with corrosion influencing at the crack tip, i.e., K_ISCC_, has to be determined for an alloy–environment system susceptible to SCC.

For valid fracture mechanics testing, it is necessary that the specimens meet the requirements of plain strain. In order to meet the requirements, the specimens for K_ISCC_ determination using traditional techniques are generally bulky, e.g., double-cantilever beam (DCB), cantilever beam (CB), compact tension (CT), centre-cracked, surface flawed (SF) test specimens [4]. Because of their bulky size and complex geometries, the fabrication of DCB, centre-cracked or CT specimens is expensive. Further, testing of such bulky specimens require high-capacity loading machines and long test times, which can be a prohibitive proposition, since the generation of reliable and statistically valid K_ISCC_ data may necessitate conducting a large number of tests. Another limitation with these specimen types is the inability to employ them when material of sufficiently thick dimensions is not available, e.g., a thin-walled component or a heat-affected zone (HAZ) in a weldment. Hence, it is extremely attractive to find a technique that can enable an accurate, cost-effective and rapid determination of K_ISCC_ using specimens of thin cross-sections (cf. DCB, centre-cracked or CT specimens).

This article reviews the determination of K_ISCC_ using the circumferential notch tensile (CNT) technique, with validation of the technique and its application through an accurate, inexpensive and rapid approach to a few industrially relevant scenarios for the generation of K_ISCC_ data.

## 2. Circumferential Notch Tensile (CNT) Testing for K_ISCC_: Distinct Advantages

**Figure 1:**
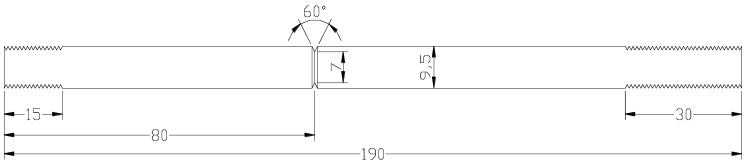
Circumferential notched tensile (CNT) specimen (all dimensions in mm) [6].

Circumferential notch tensile (CNT) specimens (shown in Figure 1) that have been used extensively in the past 15 years for the generation of valid K_ISCC_ data [1,5,6,7] circumvent all the concerns with the traditional specimen geometries (e.g., DCB, centre-cracked or CT), i.e., (a) bulky size, (b) complex geometry, (c) high-capacity loading system for achieving the loads required for large cross sections and (d) inability to test where only thin sections are available (such as HAZ of weldments or a failed thin-walled component).

The distinct advantages of a small cross section of a CNT specimen are [1,7]:

(a) It enables achieving high stress intensities (K_I_) by employing small loads;
(b) It is much easier to machine the simple cylindrical specimen geometry than the CT or DCB geometry;
(c) It is much cheaper to fabricate the specimens (cf. 20–25% of CT or DCB specimens);
(d) Enables testing when only thin sections are available (such as HAZ of weldments or a failed thin-walled component);
(e) It vastly reduces the amount of the required test material. In fact, the required test material can be further reduced considerably by using extenders. The extenders constructed out of another material can be used because the actual area of interest is only the notched portion and the adjacent area (shown in Figure 1). This enables just machining the small cylindrical sample with circumferential notch and then extending it to the full length by drilling threads on both ends (and then using extenders of a harder material to extend the length at each end);
(f) The ability to test small cylindrical samples (as described at (e) above) enables testing where only small lengths of test material may be available or where the test material is expensive (for example, when a large number of tests are required for assessing a pressure vessel constructed out of an expensive duplex stainless steel).

It is important to note CNT specimens soundly comply with the plane strain condition and low plasticity criteria in spite of their small size [8,9,10].

### 2.1. CNT Specimens for the Determination of K_ISCC_: Challenges and Circumvention

For determination of K_ISCC_, a crack growth parameter (such as time-to-fail, T_f_)), is determined at different values of stress intensity factor (K_I_) while the specimens are immersed in the corrosive fluid. K_ISCC_ is determined from the plot of T_f_ vs. K_I_. One may wonder, cylindrical geometry being so simple, why the CNT specimens were not used for the determination of K_ISCC_ until just 15 years ago. The reason lies in the complication that arises due to the eccentric nature of the pre-crack that develops during rotating–bending employed for pre-cracking of cylindrical specimen, which causes bending during tensile loading that can vastly compromise the accuracy of K_I_. Accuracy in the measurement of K_I_ is crucial for a precise determination of K_ISCC_. K_I_ of CNT specimens is determined, as described, through the Equations below [8]:

(1) $$K_{I}=\left(\sigma_{t}+\sigma_{b}\right)\sqrt{a\pi F_{O}}$$

where

(2) $$a=\frac{D-d}{2}$$

(3) $$\sigma_{t}=\frac{4P}{\pi D^{2}}$$

(4) $$\sigma_{b}=\frac{16P\varepsilon }{\pi D^{3}}$$

(5) FO=Feα(εD)

(6) $$F=\frac{1.25}{{\left[1-{\left(\frac{2a}{D}\right)}^{1.47}\right]}^{2.4}}$$

(7) α=22.188e−4.889(2aD)

The parameters in Equations (1)–(7) are: σ_t_, tensile stress (unit, Pa); σ_b_, bending stress (Pa); *F_o_*, geometric function for round specimens with eccentricity (no unit); *F*, geometric function (no unit) without eccentricity for round specimens (no unit); *a*, effective crack length (m); *D*, specimen diameter (m); *d*, equivalent ligament diameter (m); *P*, applied load (N); *ε*, eccentricity (m); *α*, a constant (no unit) calculated from Equation (7).

### 2.2. Accounting for Eccentricity (ε) in Pre-Cracked CNT Specimens

**Figure 2:**
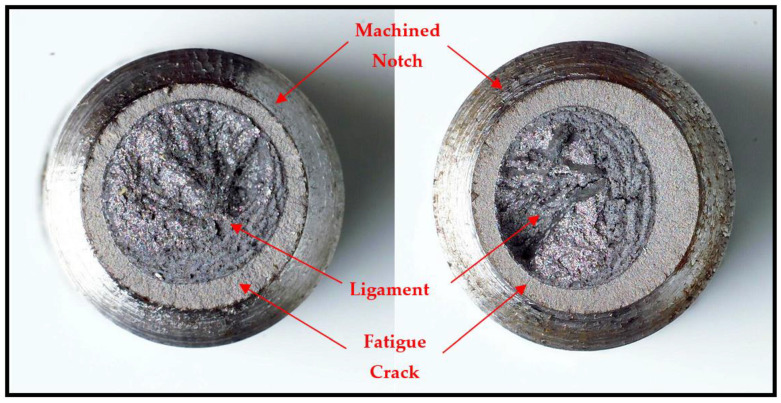
Eccentricity of fatigue crack in a CNT specimen that was subjected to rotating–bending fatigue [1].

As described earlier, the pre-crack that is introduced in a cylindrical specimen by subjecting it to rotating–bending fatigue can cause an eccentric ligament (as shown in an example in Figure 2). This eccentricity causes a critical problem in an accurate determination of K_ISCC_ by the CNT technique. As shown in Equation (1), K_I_ of specimen with an off-centre pre-crack will have a contribution of bending stress (σ_b_), in addition to tensile stress (σ_t_). Accordingly, it is essential that the bending force due to eccentricity is accounted for, for an accurate calculation of K_I_. [1]. Employing a specific method, the centroid of the eccentric ligament was determined, which enabled the determination of the bending stress experienced by the specimen due to the eccentricity. The details of the specific method can be found elsewhere [1]. In a nutshell, the effective crack length (*a*) in Equation (1) is used for the first estimate of K_I_, which enables the determination of the Irwin correction factor (r_y_) from Equation (8), and ($\bar{a}$) from Equation (9). Thus, Equation (1) enables the determination of K_I_.

(8) $$r_{y}=\frac{1}{6\pi }{\left(\frac{K_{I}}{\sigma_{Y}}\right)}^{2}$$

(9) $$\bar{a}=a+r_{y}$$

where $\sigma_{y}$ is 0.2% offset tensile yield stress (Pa). Using *a* in Equation (2), the first estimate of K_I_ is determined. $\bar{a}$ (instead of a) is used for the calculation of K_I_. The reason for this correction is described elsewhere [11,12,13,14].

### 2.3. Valid K_I_ Data

The implementation of elastic fracture mechanics (LEFM) for the determination of K_I_ assumes primarily an elastic deformation and brittle state of the material. In reality, there is invariably some contribution of plastic deformation at the crack tip before a crack propagates. Therefore, the LEFM approach allows for a small plastic zone, as long as its contribution is negligibly insignificant, which necessitates the specimen size to be large. For its implementation, two specifications have been developed for determining the validity of the measured K_I_, as described below [8,9,10]:

$$a_{f}\geq 2r_{y}\;\mathrm{and}\;\frac{\sigma_{N}}{\sigma_{Y}}\leq 2.5$$

where

$a_{f}=\varepsilon +\frac{\left(D-2a_{m}-d\right)}{2}$, and $\sigma_{N}$ is the nominal applied stress in the final ligament (Pa).

In other words, the validity requirements insist that: (i) the correction in depth due to the Irwin plastic zone (*r_y_*) should be at least half of the fatigue pre-crack depth (*a_f_*) [10,15,16] and (ii) the average of the stress across the ligament produced upon fatigue pre-cracking (*σ_N_*) should be smaller than 2.5 times the yield strength (*σ_y_*). In the context of pre-cracked CNT specimens that may have eccentric final ligament, these validity specifications can also apply (where the maximum nominal stress has contributions from both the tensile and the bending stresses which should be <2.5 *σ_y_*). Also, for such eccentric ligaments, the greatest depth of the crack is taken to be the fatigue crack depth (*a_f_*).

## 3. Procedure for the Determination of K_ISCC_ by the CNT Technique

**Figure 3:**
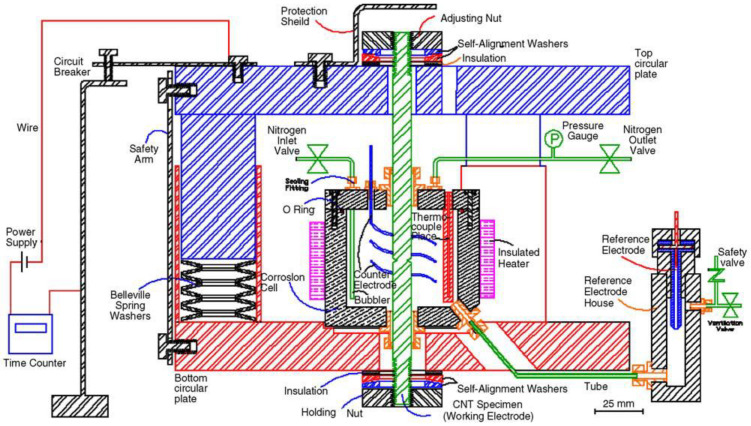
Custom-built rig for the determination of K_ISCC_ using CNT specimens (including attachments for simultaneous imposition of electrochemical potential) [6].

Figure 3 shows the diagram of the rig for CNT experiments for the determination of K_ISCC_. Besides the facility for loading the CNT specimens with the help of Bellville springs and a time counter, the rig also has a corrosion cell with facilities for: (a) the reference and the counter electrodes that enable the simultaneous imposition of electrochemical potential and (b) temperature control and thermal insulation for conducting tests with corrosive fluids at elevated temperatures. When the specimen eventually fractures, the circuit breaks, and the timer stops, enabling the determination of time-to-fail (T_f_). There is a safety protection shield at the top that stops the fractured specimen from flying away. As shown in Figure 1, the diameter of the round tensile specimen is 9.5 mm, with a 60° ‘V’ groove notch at the mid-length that reduces the diameter to 7 mm. All along the circumferential notch, a pre-crack is produced by simultaneous rotating–bending fatigue of the cylindrical specimen. However, this procedure often produces an eccentric pre-crack that can cause inaccuracy in K_I_, and its circumvention is described in the preceding section. The specimens with a pre-crack are cleaned and then loaded onto the rig. The test solution is placed into the corrosion cell. Depending on the requirement of the test, the test solution can be heated to an elevated temperature, before applying the required tensile load with the help of the Belleville springs, the adjustment nut and a tensile loading machine. The test is continued until the specimen fractures, when T_f_ is recorded. By the characterisation of the fracture surface (of the failed specimen), the eccentricity (ε) of the pre-crack is determined, following the procedure described in [1]. The determination of eccentricity (ε) enables the accurate calculation of K_I_ corresponding to the applied load, using Equations (1)–(7). The fracture surface is examined using a scanning electron microscope (SEM). Before SEM fractography, the corrosion product present on fracture surface is ultrasonically cleaned. One of the commonly used cleaning solutions contains 6 mL of concentrated hydrochloric acid + 100 mL distilled water + 10 mL of 30 g/L of 2-butyne-1,4-diol. A typical T_f_-vs.-K_I_ curve provides K_ISCC_ data; a few examples of K_ISCC_ determination are shown subsequently.

## 4. First K_ISCC_ Data Using the CNT Technique

Carbon steel in a hot caustic solution was among the first systems for which K_ISCC_ data were generated using CNT specimens [6]. SCC of steels in caustic solution is also called caustic cracking and is a vexing and age-old problem for steel vessels and pipework for Kraft process (for pulp-and-paper processing) and Bayer process (for alumina processing), which extensively employ highly corrosive caustic solutions at high temperatures and pressures [17,18,19,20,21,22,23]. For the large size of these equipment, often the affordable choice of construction material is carbon/mild steel that is known to suffer caustic cracking. Therefore, caustic cracking is a critical consideration for the design of such equipment, as well as for its in-service maintenance, safety and life extension/prediction. Since caustic chemistry and temperature vary considerably across a plant and across industry, there may be the need of generating a huge amount of K_ISCC_ data. In this respect, CNT testing comes as an attractive option for the cost-effective and rapid generation of K_ISCC_ data. The determination of K_ISCC_ of carbon steel for one combination of caustic composition and temperature is described here.

**Figure 4:**
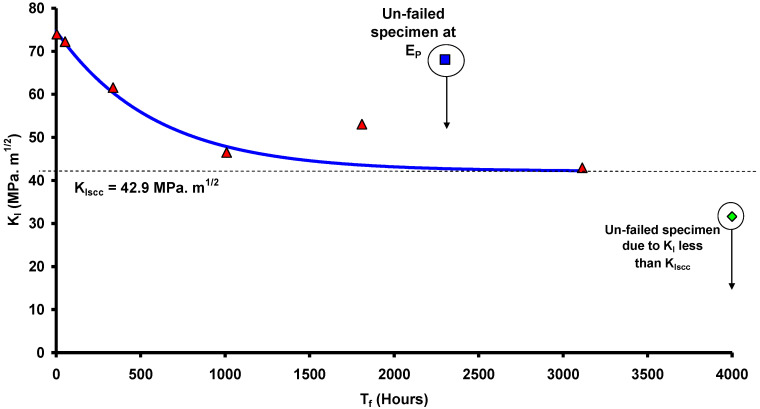
K_I_-vs.-T_f_ plot for CNT specimens of carbon steel in a caustic solution (500 g/L, 100 °C) tested at open circuit potential (the “Un-failed specimen” was tested at a passivating imposed potential, E_p_) [5].

Employing the testing rig shown in Figure 3, CNT specimens (Figure 1) were tested at various magnitudes of K_I_ of carbon steel immersed in a caustic solution (500 g/L at 100 °C) that produced a plot of K_I_ vs. time-to-failure (T_f_), as shown in Figure 4. As seen from Table 1, only those values of K_I_ that satisfied the validity requirements were used for generating the plot in Figure 4 (as described Section 2.3). The value of K_I_ that produced a horizontal asymptote to T_f_ axis is K_ISCC_. As shown in Figure 4, this procedure determined the K_ISCC_ of carbon steel immersed in the 500 g/L caustic solution at 100 °C to be ~43 MPa·m^1/2^. Accordingly, in the specimen that was tested at a K_I_ value below K_ISCC_ (i.e., ~32 MPa·m^1/2^), the crack did not propagate even after 4000 h of testing, and the specimen did not fail.

**Figure 5:**
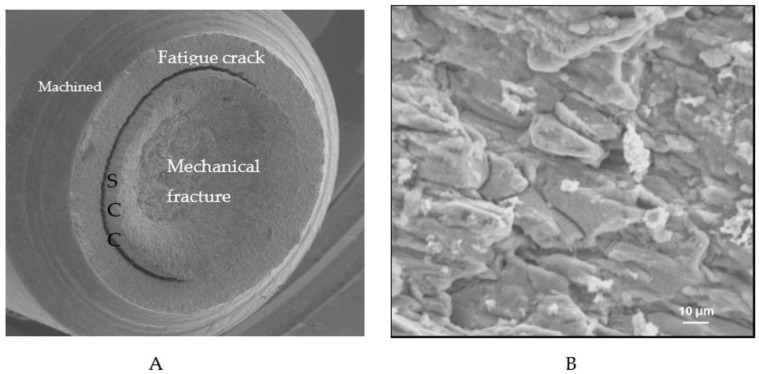
Representative SEM fractographic feature of CNT specimens (tested in 500 g/L NaOH solution at 100 °C), with K_I_ > K_ISCC_: (**A**) entire fracture surface, with areas of notch, fatigue pre-crack, SCC and the central area of overload “Mechanical fracture” and (**B**) magnified area immediately ahead of the pre-crack (identified as ‘SCC’ in Figure 5A), suggesting the crack propagation to be intergranular [5].

Figure 5A is a representative fractograph showing different areas with distinct fractographic features for specimens that were tested at K_I_ > K_ISCC_ (i.e., 43 MPa·m^1/2^). The higher magnification image of the fractographic area immediately ahead of the pre-crack (Figure 5B) suggests the crack to have propagated in intergranular mode, which is distinctive evidence of SCC/caustic cracking [5].

### 4.1. CNT Testing under Imposed Electrochemical Condition

Figure 4 also includes a data point at a K_I_ of 68 MPa·m^1/2^ for a specimen tested at an imposed electrochemical potential in the passivating regime (E_p_) of +278 mV. As the plot in Figure 4 would suggest, in the absence of the imposed potential, a specimen at the K_I_ of 68 MPa·m^1/2^ should have failed in just ~200 h, whereas, with the imposed potential of +278 mV, the specimen did not suffer failure even after 2300 h of testing. This observation indicates the possibility of imposition of a suitable electrochemical potential to retard the propagation of the stress corrosion crack in industrial situations, and it is interesting to understand the mechanistic basis for such retardation.

**Figure 6:**
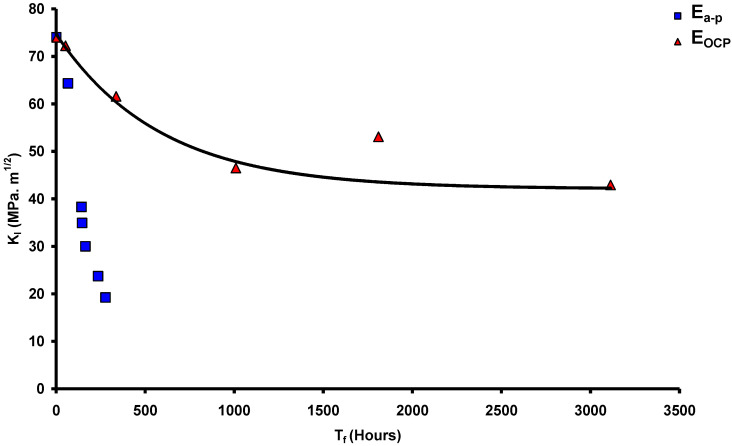
Comparison of K_I_ and T_f_ plots for CNT specimens of carbon steel in a caustic solution (500 g/L/100 °C) tested at the open circuit potential (OCP) and an imposed active–passive potential (E_a-p_) [5].

A breakdown of a passive film that enables rapid localised dissolution is the most accepted mechanism for stress corrosion crack propagation [5]. A passive film develops on the entire surface including at the tip of the existing pre-crack. However, the passive film suffers the localised rupture at the crack tip because of a high local stress intensity. This rupture creates a narrow anodic area where rapid dissolution occurs, which amounts to propagation of the stress corrosion crack. However, the tip repassivates, but the passive layer again suffers localised cracking. The repetition of the cycle of dissolution and repassivation sustains the growth of the stress corrosion crack. However, if the crack tip were to develop too robust a passive film that could not be ruptured by the local stress intensity, then crack cannot grow. A robust passive film can be achieved by imposition of highly passivating electrochemical conditions. This was the case when a highly passivating electrochemical potential (E_p_) of +278 mV was imposed on a carbon steel specimen tested at a K_I_ value of 68 MPa·m^1/2^, and as a result of this strongly passivating condition, the specimen did not suffer failure even after 2300 h of testing (Figure 4). In the absence of such passivating potential, the specimen tested at 68 MPa·m^1/2^ would have failed in ~200 h, as Figure 4 suggests. A confirmation to this mechanistic role of passivating imposed potential in the retardation of SCC is the acceleration in crack propagation when the imposed potential constitutes a condition of transition between active and passive potentials (E_a-p_). The regime of active–passive transition potential (E_a-p_) for carbon steel in a 500 g/L caustic solution at 100 °C was identified by anodic polarization experiments [5]. The potential of −723 mV, that is within the E_a-p_ regime, was imposed on the specimens during CNT tests at a few K_I_ values using a 500 g/L NaOH solution at 100 °C. One can appreciate that this imposition of E_a-p_ strongly facilitates the condition for SCC through a passivation–dissolution–repassivation mechanism and thereby, it should accelerate the failure. Indeed, a comparison of K_I_-vs.-T_f_ data, with and without imposed potential, as in Figure 6, shows remarkable acceleration in failure under the imposed E_a-p_ condition.

## 5. Validation of the CNT Testing Technique for K_ISCC_ Data Generation

With the remarkable advantages of the CNT technique for conducting fracture mechanics-based tests for the generation of K_ISCC_ data as well as its comprehensive implementation for the generation of K_ISCC_ data for caustic cracking of carbon steel (as described in Section 4), it was prudent to investigate its validity vis-à-vis the established techniques (e.g., DCB or CT testing). Since K_ISCC_ data for 4340 steel in an aqueous chloride environment available in the literature [24,25] were generated using other techniques, this material was selected for generating K_ISCC_ data using the CNT technique, for the purpose of comparison of the data from different techniques, thereby investigating the validity of the CNT technique [7].

**Figure 7:**
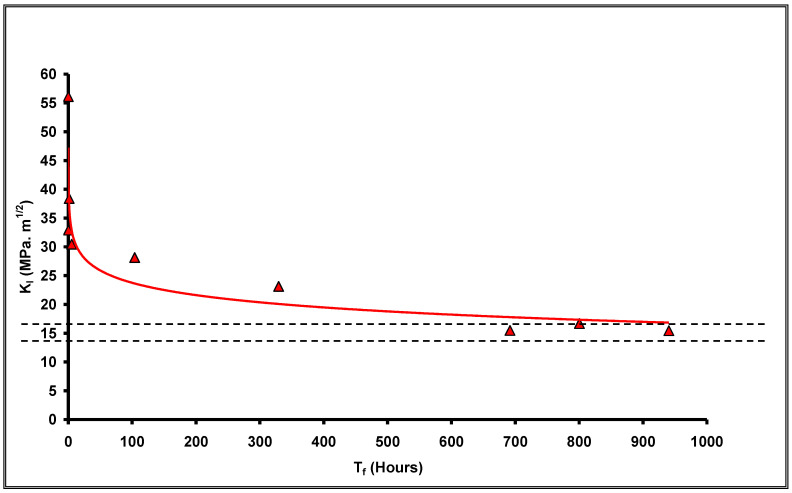
Plot of K_I_ vs. T_f_ for 4340 steel in 0.1 N chloride solution [7].

Following the procedures described in Section 3 and Section 4, CNT specimens of 4340 steel were tested in 0.1 N NaCl (i.e., similar to the solutions used in earlier studies [24,25]). ‘Cantilever bend’ [24] and ‘centre cracked’ [25] were the specimen types employed in earlier studies. From the plot of K_I_ vs. time-to-failure (T_f_) (Figure 7), K_ISCC_ of 4340 steel was determined to be ~15 MPa·m^1/2^. K_ISCC_ of this steel in 0.1N NaCl was reported [24,25] to be 14–16 MPa·m^1/2^, as shown in the band in Figure 7. Thus, K_ISCC_ of 4340 steel determined using CNT specimens (~15 MPa·m^1/2^) is within this band, which validates the CNT technique for the accurate determination of K_ISCC_.

## 6. K_ISCC_ of Narrow Structures: Determination by the CNT Technique

**Figure 8:**
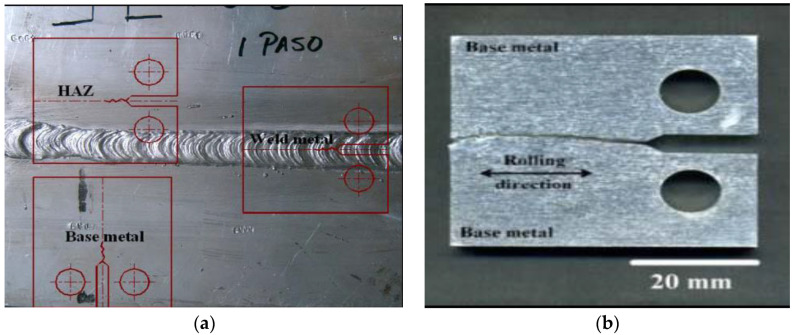
(**a**) Schematic of the extraction of CT specimens from microstructurally different areas of a steel weldment, i.e., weld metal, HAZ and base metal, location of the notch in each case, and (**b**) out-of-plane propagation of the crack.

As elaborated in Section 1, the traditional techniques for the determination of K_ISCC_ (e.g., CC, SF, CB, DCB and CT) have a mandatory requirement of bulky specimens to satisfy the plane strain condition. There have been attempts to test CT specimens of narrow regions such as by extracting specimens of a heat-affected zone (HAZ) or weld metal from the weldment and then creating a notch/pre-crack right in the location of interest (as shown in Figure 8a). However, this approach is fraught with the untoward possibility of the out-of-plane propagation of the crack into the adjacent areas (as shown in Figure 8b) and hence inaccuracy in the data. Out-of-plane crack propagation is particularly possible for very narrow structures such as an HAZ, which is the most critical region to be tested, since an HAZ is often most susceptible to mechanical and corrosion-assisted failures. The diameter of CNT specimens of steels can be as narrow as 7 mm, which circumvents the limitations due to the bulky nature of traditional specimens, as well as due to the geometrical constrains restricting the cracks within the plane of the notch.

**Figure 9:**
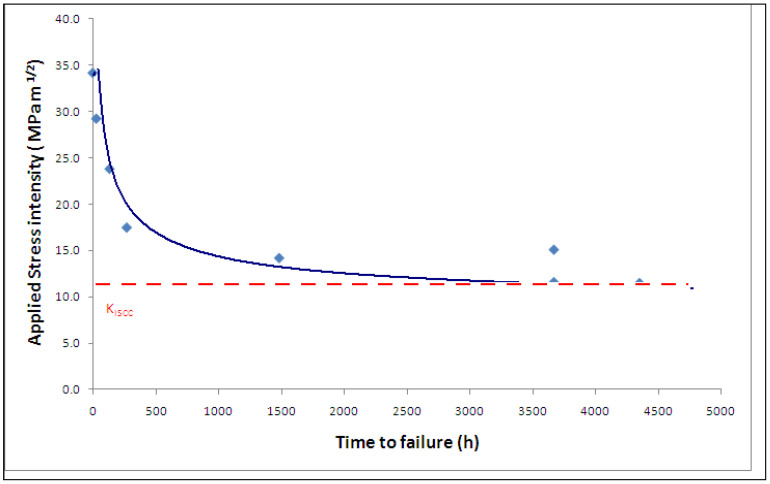
Plot of K_I_ vs. time-to-failure for weld metal extracted from a steel weldment [26].

For the determination of K_ISCC_ of steel weld, plates of 250 grade steel were welded. CNT specimens were machined perpendicular to the weld direction, with a notch within the weld [26]. Tests on CNT specimens of weld were carried out in 30 wt.% NaOH at 100 °C by the procedure described in Section 4. As shown in Figure 9, K_ISCC_ was determined to be 11 MPa·m^1/2^. The ability to determine K_ISCC_ of weld validates the capability of the CNT technique to test a narrow section.

**Figure 10:**
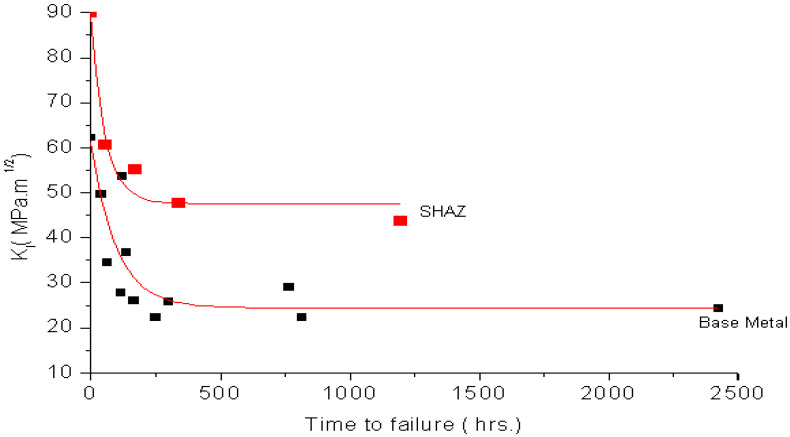
Comparison of K_ISCC_ of simulated HAZ (SHAZ) and base metal of grade 250 steel in a caustic solution (30 wt.%) at 100 °C [27].

Another approach for generating K_ISCC_ data for a narrow structure with a distinct microstructure (such as HAZ or weld metal) is to simulate that microstructure in the entire specimen by subjecting it to the suitable thermal treatment. In principle, this approach can be adopted for traditional specimen types (such as CT or DCB), but, again, CNT specimens have a distinct advantage because of their cylindrical geometry which is considerably more suitable for achieving symmetrical thermal treatment and development of a uniform microstructure (such as that obtained by using a Gleeble thermal simulator). The HAZ experiences a given thermal cycle during welding of 250 grade steel that was simulated in CNT specimens, using a Gleeble thermal simulator [27]. It is important to note that the relevant microstructure needs be replicated only in the limited section of the CNT specimen where a notch can be easily produced. CNT specimens of simulated HAZ and base metal were tested in 30 wt.% NaOH at 100 °C by the procedure described in Section 4. K_ISCC_ of simulated HAZ and base metal were respectively determined to be ~45 MPa·m^1/2^ and 24 MPa·m^½^ (Figure 10) [27]. It is interesting that HAZ showed higher K_ISCC_ (i.e., greater resistance to stress corrosion crack propagation), which is inconsistent with the common observation of inferior properties of HAZ; however, the explanation for this behaviour, which is outside the scope of this article, can be found elsewhere [27].

## 7. CNT Testing to Generate Data for Other Commercial Applications

### 7.1. SCC Resistance of Biodegradable Implant Materials

**Figure 11:**
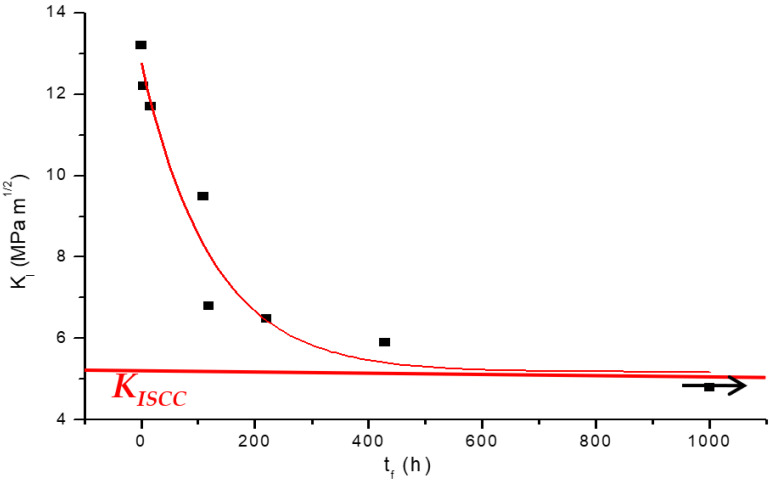
K_I_-vs.-t_f_ plot for CNT specimens of a Mg alloy (AZ91D) tested at ~36 °C in a modified simulated body fluid (m-SBF) [33].

Magnesium alloys are attracting increasing interest for their innovative application as temporary biodegradable implants, e.g., plates, wires, screws, stents etc. [28,29,30,31,32]. It may be intriguing that in such an application, the high corrosion susceptibility of magnesium and its nontoxic nature are exploited in the sense that Mg alloy implants can harmlessly dissolve away in the human body, after the device has performed its temporary function. A surgery is invariably carried out to remove the temporary implant when it is constructed out of a tradition material (such as Ti alloys). This second surgery can be entirely avoided if such implants could be constructed out of Mg alloys [30,31,32,33]. Such implants invariably possess sharp contours that can act as stress intensity points for the initiation of stress corrosion cracks. Therefore, it is prudent to understand the susceptibility of an Mg alloy to SCC and generate K_ISCC_ data for the proper design of the implant. Employing the procedure and CNT specimens described in Section 4, K_ISCC_ of a Mg alloy was determined to be 5.2 MPa·m^1/2^ in a simulated body fluid (SBF) at 36 °C (Figure 11) [33].

### 7.2. Improved Caustic Cracking Susceptibility Diagrams

**Figure 12:**
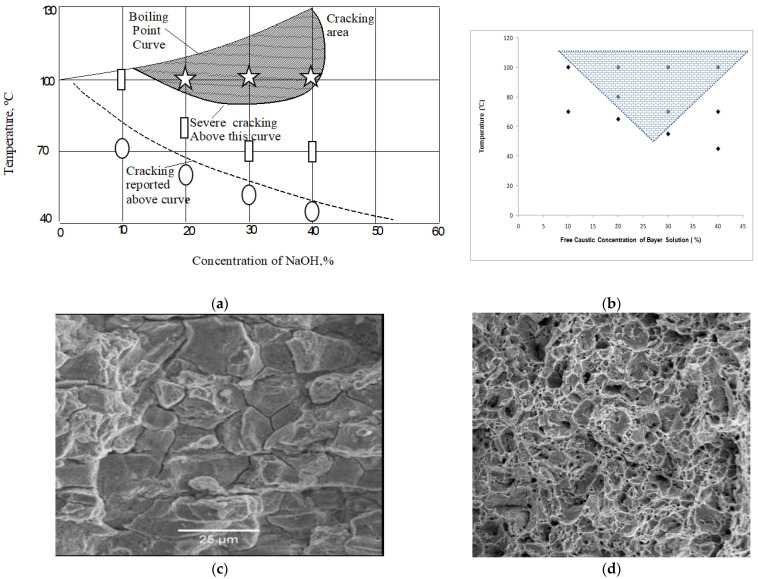
(**a**) CS diagram in a plain caustic solution superimposed with some data generated using CNT testing. CNT data: the stars represent the region of definite cracking, the rectangles represent the region of likely cracking, and the ellipses represent the region of definite absence of cracking [34]. (**b**) CS diagram in Bayer solutions, generated using CNT specimens showing caustic cracking susceptibility regions. The hatched area on the diagram represents the regime of caustic cracking susceptibility, and the points represent the conditions of the actual tests [34]. (**c**) Representative SEM fractograph of areas showing intergranular caustic cracking [38]. (**d**) Representative SEM fractograph showing ductile dimples that represent exclusively mechanical failure (devoid of caustic cracking) [38].

Section 4 describes the stress corrosion cracking of steels in an alkaline solution (i.e., caustic cracking) as a serious concern in pulp-and-paper processing (by Kraft process) and alumina processing (by Bayer process). For example, for extracting alumina from ore (bauxite), aggressive caustic solutions are extensively used at high temperatures and pressures in digesters, decomposer and precipitator in the Bayer process. Temperature and composition of caustic solutions vary vastly across these pieces of equipment in a given plant as well as across industry [34,35,36,37,38]. It is crucial to have plant-relevant data on the thresholds of caustic concentration and temperatures for causing caustic cracking to steels, since reaction vessels and pipes are most commonly constructed from steels [17,18,19,20,21,22]. On the basis of laboratory and industrial tests for up to 62 days at different caustic concentrations and temperatures, caustic cracking susceptibility (CS) diagrams (e.g., Figure 12a [34]) have been developed. Such diagrams are used for the design and operation of alumina processing plants. The upper hatched area in the diagram in Figure 12a represents the regime of severe cracking, whereas it is uncertain whether caustic cracking will occur in the regime below the hatched area. For example, the CS diagram suggests that steels in 15–40 wt% NaOH is most likely to suffer SCC at temperatures >95 °C, but cracking may or may not take place at temperatures <80 °C. The upper hatched area was constructed upon laboratory tests, whereas the field data were the basis for the construction of the lower curve [34,35].

As stated earlier, there is ambiguity about susceptibility for the part of the diagram that lies between the upper and the lower regimes, and an improvement in this domain will translate into the ability for a more accurate life prediction/extension of the equipment. Other difficulties with the CS diagram shown in Figure 12a are:

(a) The diagram was developed using plain NaOH solutions, whereas the actual Bayer liquors or Kraft solutions have several impurities. Some of the impurities have strong influence on the chemical and physical characteristics of the passive films. Given that the susceptibility to SCC is profoundly dictated by the nature of the corrosion/passive film at the tip of a crack (as elaborated at Section 4.1), the susceptibility to caustic cracking is likely to be influenced by the chemical variations of caustic solutions (such as those due to impurities). For example, a minor increase in sulphur content enhances susceptibility to caustic cracking, as does the addition of aluminates ions (AlO_2_^−^) [17]. Hence, it is essential to develop separate CS diagrams for Bayer liquors or Kraft solutions with different chemistries. However, this will require a large number of tests, necessitating the employment of a cost-effective testing technique.
(b) Smooth samples of steel were used for developing the diagram [35,36,37], whereas the fabricated components often have sharp notches such as machining burs and other stress raisers such as surface defects. Therefore, it is prudent to investigate the validity of the caustic susceptibility diagram by testing notched and pre-cracked specimens [34,38].

The requirements described in the preceding paragraphs necessitate a large number of tests on pre-cracked specimens. Its simplicity and inexpensive nature make CNT testing extremely attractive for a large number of tests. Using the CNT technique and typical Bayer liqueur solutions of various free caustic compositions at different temperatures as described in Table 2, CNT tests were conducted on steel samples. These tests resulted in the development of a model caustic cracking susceptibility diagram, as shown in Figure 12b. Table 2 also summarizes the fractographic evidence for the caustic cracking and its absence in various specimens corresponding to the CS diagram in Figure 12b. Figure 12c is a representative fractograph showing that the crack propagated by intergranular mode, i.e., a confirmatory feature of caustic cracking that occupied a considerable fraction of the fracture surface of such samples (as identified in Table 2). On the other hand, as shown in their representative fractograph in Figure 12d, in the case of samples that did not suffer caustic cracking (as identified in Table 2), the entire fracture surface was occupied by ductile dimples (and such samples were devoid of the intergranular mode of cracking seen in Figure 12c). The diagram shown in Figure 12b may serve as an improved guideline for monitoring and mitigation of the continuing concerns of caustic cracking in the Bayer’s process of alumina processing industry.

### 7.3. SCC of SG Cast Iron Equipment for Alumina Processing

**Figure 13:**
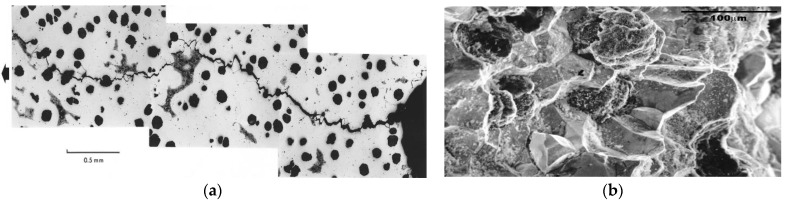
Caustic cracking observed in a failed in-service equipment from alumina processing line constructed from cast iron: (**a**) montage of optical micrographs tracking the propagation of caustic crack and (**b**) SEM fractograph of the fracture surface of the caustic crack seen in Figure 14a, evidencing intergranular propagation [39].

In one of the early studies on the exploration of the use of CNT specimens, K_ISCC_ of a cast iron was determined in caustic solutions at elevated temperatures [6,23]. Cast iron was chosen for this early study because it is inherently brittle and hence, it easily complies with the constraints required for the implementation of linear elastic fracture mechanics (LEFM). Also, SG cast iron is another commonly used material of construction of equipment for alumina processing, where hot caustic solutions are extensively used. Cast iron suffers caustic cracking in such use [39], as shown through the in-service caustic cracking of equipment constructed from cast iron (Figure 13a,b).

**Figure 14:**
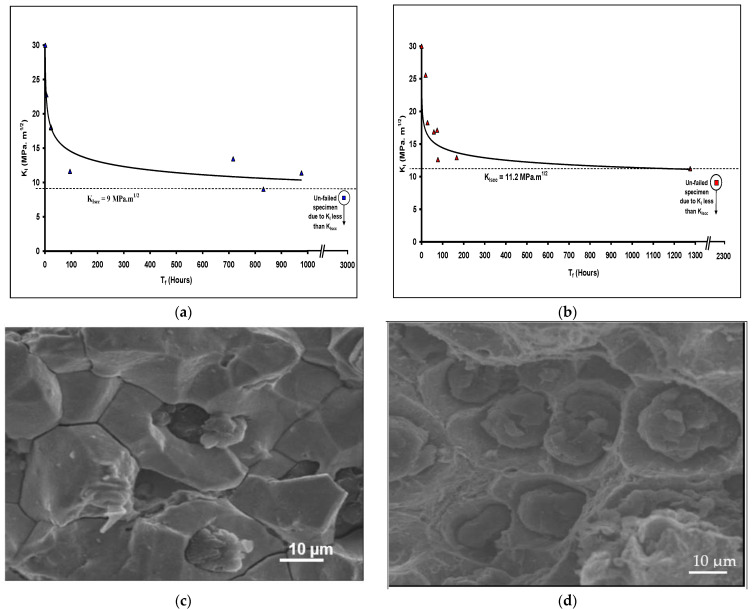
Caustic cracking of CNT specimens of SG cast iron tested in a caustic solution (200 g/L): (**a**) K_I_-vs.-T_f_ plot at 120 °C, (**b**) K_I_ -s.-T_f_ plot at 100 °C, (**c**) SEM fractograph showing intergranular caustic cracking and (**d**) SEM fractograph showing exclusively mechanical failure [1].

Employing the procedure and CNT specimens described is Section 4, K_ISCC_ of cast iron in 200 g/L was determined to be 9 MPa·m^1/2^ (Figure 14a) and 11 MPa·m^1/2^ (Figure 14b) at 100 and 120 °C, respectively [1,6,23]. SEM confirmed intergranular caustic cracking in the area areas of SCC (Figure 14d) as well as exclusively mechanical failure and graphite nodules over the rest of the fracture surface (Figure 14d).

### 7.4. Chloride SCC of Sensitized Stainless Steel

Vessels and pipes for equipment handling corrosives in chemical processing plants as well as structural members in marine construction and power plants are commonly constructed from austenitic stainless steels that possess a combination of the required properties, i.e., strength and toughness, corrosion resistance, weldability and fabricability. However, when an austenitic stainless steel (SS) with sufficient carbon content (>0.3 wt.%) is heated for sufficient time at 415–850 °C, its microstructure undergoes sensitization, i.e., extensive formation of chromium carbide (Cr_23_C_6_) precipitates at the grain boundaries that causes concurrent depletion of free Cr in the area adjoining grain boundaries. Such Cr-depleted areas fail to passivate adequately and suffer rapid intergranular corrosion (IGC) when exposed to an aqueous chloride environment. Because the heat-affected zone (HAZ) of weldments of austenitic SS experiences a temperature regime of 415–850 °C for considerable durations during welding, HAZ undergoes extensive sensitization and suffers rapid IGC in a corrosive environment. This type of corrosion-assisted degradation of welds is called weld decay, which is a major concern for the mechanical integrity of welded equipment of austenitic SS operating in a corrosive environment. This concern is further accentuated if sufficient stresses are present, which, in synergy with corrosion, can cause intergranular stress corrosion cracking (IGSCC). Therefore, it is of utmost importance to generate K_ISCC_ data for HAZ in the environment of interest, such as an aqueous chloride solution. As described in Section 6, it is prohibitively difficult to generate K_ISCC_ data for small samples such as the narrow region of HAZ by the traditional techniques that require bulky samples in order to meet the plane strain condition. Section 6 also describes how CNT specimens meet the plane strain condition in spite of their relatively narrow cross section.

**Figure 15:**
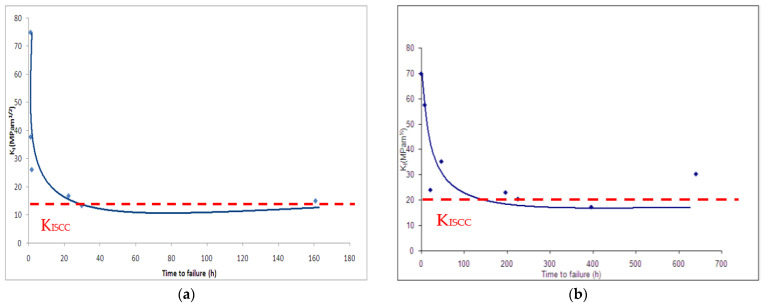

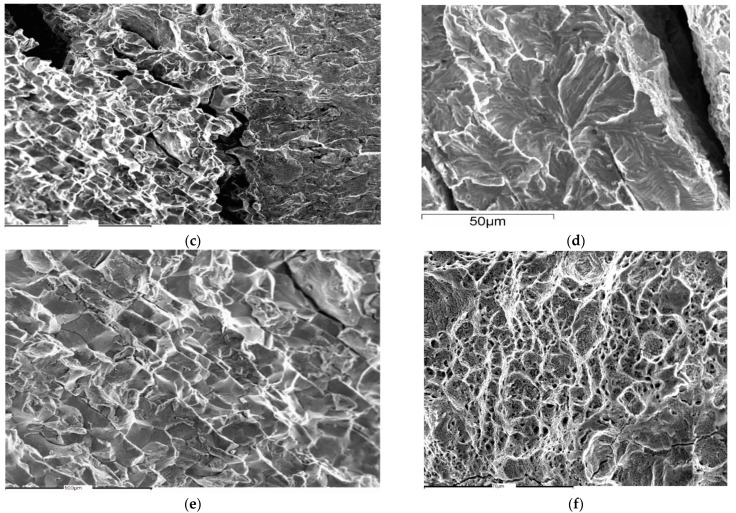
CNT testing of 304 austenitic stainless steel (SS) in 42 wt.% MgCl_2_ at 154 °C: (**a**) K_I_-vs.-T_f_ plot for sensitized SS, (**b**) K_I_-vs.-T_f_ plot for SS base metal (unsensitised), (**c**–**f**) representative SEM fractographs: (**c**) region of mixed-mode SCC (transgranular and intergranular), (**d**) region of only transgranular SCC, (**e**) region of only intergranular SCC and (**f)** central area with dimples that suggest exclusive mechanical failure [41].

A sensitized microstructure was developed in CNT specimens of type 304 austenitic SS [40,41]. Employing the procedure described is Section 4, the CNT technique determined K_ISCC_ of the sensitized austenitic SS in 42 wt.% MgCl_2_ at 154 °C to be 11 MPa·m^1/2^ (Figure 15a), whereas K_ISCC_ of the base metal (i.e., without sensitization) under the identical condition was 16 MPa·m^1/2^ (as shown in Figure 15b) [40]. SEM fractographs of both sensitized and unsensitised SS possessed distinct features of stress corrosion cracking (SCC), i.e., intergranular SCC (IGSCC) and transgranular SCC (TGSCC), as seen in Figure 15c–e, and those of mechanical overload fracture (i.e., the dimples suggesting ductile failures, as shown in Figure 15f) in the central area. However, features of SCC (i.e., IGSCC and TGSCC) occupied a greater fraction of the fracture surface of sensitized SS.

## 8. Conclusions

1. Distinct advantages of circumferential notch tensile (CNT) specimen geometry in generating critical stress corrosion cracking (SCC) data because of their small cross-sections are:
  (a) It is much cheaper to fabricate CNT specimens than specimens with most of the traditional geometries (e.g., CT or DCB), which enables conducting a large number of fracture mechanics-based tests on pre-cracked specimens that are necessary for the generation of quality K_ISCC_ data. It also is easier to machine specimens with a simple cylindrical geometry.
  (b) The considerably smaller cross-sectional area enables achieving high stress intensities (K_I_) by using moderate loads.
  (c) The amount of the required test material is vastly smaller, and it is possible to perform tests when only thin sections are available (such as HAZ of weldments or a failed thin-walled component). This is also advantageous when the test material is expensive.
2. Because of the advantages described above, CNT testing is a relatively simple and inexpensive technique for the generation of data for the design, maintenance and life assessment of equipment susceptible to SCC, such as SCC of notched specimens or K_ISCC_ data.
3. The CNT technique has been successfully validated and employed for characterising the SCC susceptibility of notched specimens and/or for generating K_ISCC_ data for various commercial scenarios, such as caustic cracking of steels and cast iron, and can be used to develop more relevant cracking susceptibility diagrams. CNT techniques has also been employed for generating crucial SCC data for bioimplant materials, narrow regions of steel welds and heat-affected zone and sensitized stainless steel.

## Figures and Tables

**Table 1:** Experimentally determined K_I_ data and proof of the K_I_ data meeting the validity requirements (refer Section 2.3) for carbon steel at open circuit potential and an imposed passive potential (E_p_) in 500 g/L NaOH at 100 °C.

K_I_ (MPa·m^1/2^)	T_f_ (hours)	$a_{f}\;(\mathrm{mm})$	2r_y_ (mm)	$\frac{\sigma_{N}}{\sigma_{Y}}$
74 *	0.5	1.32	1.20	2.39
72.2	53	1.56	1.16	1.91
61.6	337	1.29	0.89	2.03
46.5	1009	0.90	0.53	1.44
53.1	1810	0.93	0.67	1.51
42.9	3113	0.66	0.46	1.41
31.6 ^$^	4006 ^@^	1.25	0.27	1.06
68.1 ^#^	2301 ^@^	1.26	1.06	2.15

* K_IC_ (fracture toughness), ^$^ Un-failed specimen due to K_I_ < K_ISCC_, ^@^ Test duration, no failure. ^#^ Specimen tested at E_p_ (Un-failed).

**Table 2:** Investigation of caustic cracking in different Bayer solutions by CNT testing and resulting fractographic features [38].

Free Caustic Concentration, wt%	Caustic Solution Temperature, °C	Applied Stress Intensity, MPa·m^1/2^	Time to Failure, h	Evidence of Intergranular Fractographic Features	Caustic Cracking Susceptibility Feature
10	100	37.3	2088 h	Yes	Yes
	70	33.1	Did not Fail in 2323 h	No (Forced to Fracture)	No
20	100	31.7	241 h	Yes	Yes
	80	27.3	2280 h	Yes	Yes
	80	32.3	950 h	Yes	Yes
	65	62.9	Did not Fail in 4000 h	No (Forced to Fracture)	No
30	100	27.3	1801 h	Yes	Yes
	70	42.2	87 h	Yes	Yes
	55	53.2	210.3 h	Yes	Yes
40	100	75.1	192 h	Yes	Yes
	70	41.2	Did not Fail in 2112 h	No (Forced to Fracture)	No
	45	30	Did not Fail in 1632 h	No (Forced to Fracture)	No

## Data Availability

Data are contained within the article.
